# Correlations between IL-36 family cytokines in peripheral blood and subjective and objective assessment results in patients with allergic rhinitis

**DOI:** 10.1186/s13223-023-00834-y

**Published:** 2023-08-30

**Authors:** Jia Gu, Gang Qin, Liang Jiang, Wei Xu, Yuanyuan Wang, Jiangxue Liao, Hongzhu Pan, Zhuoping Liang

**Affiliations:** Department of Otolaryngology Head and Neck Surgery, The Affiliated Hospital of Southwest Medical University, Taiping Street & NO. 25, Luzhou, 646000 Sichuan China

**Keywords:** Allergic rhinitis, Interleukin-36, Symptom assessment, Allergens

## Abstract

**Background:**

Interleukin (IL)-36 family cytokines have received increasing attention, especially in the fields of inflammation and immunity research. However, whether IL-36 family cytokine levels are correlated with the results of the assessment of allergic rhinitis (AR) and affect the severity of AR remains unknown. Therefore, this study aimed to investigate the correlations between IL-36 family cytokine levels and subjective and objective assessment results and to further analyze the possible mechanisms of IL-36 family cytokines in the development of AR.

**Methods:**

An enzyme-linked immunosorbent assay (ELISA) was used to detect the concentrations of the IL-36 family cytokines IL-36α, IL-36β, IL-36γ, IL-36Ra, and IL-38 in the peripheral blood of patients with AR. The condition of patients with AR was assessed by 22-item sino-nasal outcome test (SNOT-22) score, visual analogue scale (VAS) scores for disease severity, and serum inhalant allergen immunoglobulin E (IgE) detection. Correlations between IL-36 family cytokine levels and subjective and objective assessment results in patients with AR were analyzed.

**Results:**

The concentration of IL-36α in the peripheral blood of patients with AR was the highest, and the concentration of IL-36β was the lowest. The concentration of IL-36α was higher in juvenile patients than in adult patients, and there was a difference in the IL-36Ra level between the perennial allergen group and the seasonal allergen group. There was a positive correlation between IL-36α level and IL-36γ level, IL-36γ level and IL-36Ra level, and IL-36Ra level and IL-38 level, and IL-36β level was positively correlated with IL-36Ra and IL-38 levels, respectively. IL-36α level was positively correlated with VAS score for nasal congestion symptom. IL-36β level was positively correlated with the total VAS score for ocular symptoms and VAS scores for ocular itching and eye pain symptoms. However, there was no correlation between the levels of all cytokines in IL-36 family and SNOT-22 score, the number of positive inhaled allergens, or the highest positive intensity of allergen specific immunoglobulin E (sIgE).

**Conclusion:**

Peripheral blood IL-36 family cytokines play an important role in AR, and the concentrations of IL-36α and IL-36β were related to the severity of symptoms in patients with AR.

## Introduction

Allergic rhinitis (AR) is an allergic respiratory disease mediated by immunoglobulin E (IgE) after body contact with allergens and is mostly characterized by nasal itching, sneezing, runny nose, and nasal congestion. AR is a global disease with a high incidence, affecting 20–30% of adults and up to 40% of children in the United States and Europe [1]. Studies have shown that the risk factors of AR mainly include demographic factors, genetic factors, air pollution, and environmental allergen exposure [2]. The corresponding symptoms and signs produced by the allergen after continuous or repeated stimulation of the body lead to fatigue, mood changes, impaired cognitive function, and even depression and anxiety, thus seriously affecting the quality of life of patients [3].

Interleukin (IL)-36 family cytokines are encoded by a gene located on human chromosome 2. Its structure closely resembles that of the classic interleukin (IL)-1 superfamily [4], of which it is considered a novel member, and the IL-36 family includes IL-36α, IL-36β, IL-36γ, IL-36Ra, and IL-38 [5]. As inflammatory agonists, IL-36α, IL-36β, and IL-36γ bind to the IL-36 receptor (IL-36R) and recruit the coreceptor interleukin-1 receptor accessory protein (IL-1RAcP), which in turn activates myeloid differentiation response gene 88 (MyD88), leading to activation of the nuclear factor-κB (NF-κB) and mitogen-activated protein kinase (MAKP) pathways, which in turn produce many proinflammatory cytokines and chemokines and induce inflammatory responses [6]. In contrast, IL-36Ra and IL-38 act as antagonists of IL-36R when combined with them, block intracellular signaling and inhibit inflammatory responses [6–8].

As a newly described member of the IL-1 superfamily, IL-36 has received growing attention. Increasing evidence suggests that IL-36 family cytokines are key mediators of a variety of inflammatory diseases and are closely related to development of diseases [9, 10]. Patrick et al. [11] found that patients with atopic dermatitis (AD) had elevated levels of IL-36α and IL-36γ in peripheral serum, and IL-36α promoted IgE production by B cells in vitro through activation of IL-36R. Recent studies have shown [12] that IL-36Ra is expressed at lower levels in the peripheral serum of asthmatic patients than in healthy controls and that IL-36Ra can suppress the inflammatory response in mouse models of asthma. In addition, abnormally expressed IL-36 family cytokines were detected in patients with chronic rhinosinusitis with nasal polyps (CRSwNP), the refractory form of which is believed to be associated with increased IL-36α concentration as a risk factor [13], while IL-36γ promoted the secretion of chemokines and adhesion factors and induced neutrophil infiltration [14]. IL-36α, IL-36β, and IL-36γ, which are highly expressed in the sinus mucosa of patients with chronic rhinosinusitis (CRS), can act as response elements to microorganisms and other organisms through Toll-like receptor (TLR) signaling pathways and promote CXC class chemokine production to interact with innate and adaptive immune responses in CRS [15]. Significantly higher protein concentrations of IL-36 family cytokines were also detected in the peripheral serum of patients with AR than in normal controls, suggesting that IL-36 family cytokines may be involved in the pathogenesis of AR [16], but the relationship between IL-36 family cytokines and disease severity in patients with AR is unknown.

In summary, this study is the first to correlate the levels of IL-36 family cytokines in the peripheral blood of patients with AR with subjective and objective assessment results to further investigate the possible role of IL-36 family cytokines in the pathogenesis of AR and their relationship with the severity of AR, thereby providing new ideas for studying the action mechanisms of IL-36 family cytokines in AR and new targets for AR treatment.

## Methods

### Study subjects

Seventy-seven patients with AR (20 patients younger than 18 years old, including 10 males and 10 females, and 57 patients aged 18 years old or older, including 23 males and 34 females) who were admitted to our Otolaryngology Head and Neck Surgery clinic between January 2022 and June 2022 were selected for the study. This study was reviewed and approved by the Medical Ethics Committee of the Affiliated Hospital of Southwest Medical University, and informed consent was obtained from all patients (patients younger than 18 years old were given informed consent signed by their legal guardians). The inclusion criteria were as follows: (1) Relevant diagnostic criteria for AR in Allergic Rhinitis and its Impact on Asthma (ARIA) guidelines — 2016 revision [17]; (2) Positivity for at least one allergen test per serum specific immunoglobulin E (sIgE) ; (3) Complete clinical data; (4) Age > 5 years, male or female. The exclusion criteria were as follows: (1) Combined with nasal polyps, severe nasal septum deviation, sinusitis, atrophic rhinitis, or other nasal disease; (2) Combined with immunodeficiency disease, malignant tumors, cardiovascular disease, mental illness and important organ dysfunction, or other serious wasting disease; (3) Combined with respiratory tract or other allergic diseases; (4) Use of local, systemic glucocorticoids, antihistamines or immunotherapy in the past month; (5) Age ≤ 5 years; (6) Participation in other medical studies at the same time.

### Study methods

#### Serum specimen collection

Five milliliters of peripheral venous blood was drawn from patients with AR and injected into sterile tubes containing anticoagulant heparin, allowed to stand at room temperature for 20–30 min, and centrifuged at 3000 r/min for 10 min; then, the upper layer of serum was aspirated and transferred into 2 ml cryopreservation tubes (in duplicate to prevent repeated freezing and thawing) and placed in a -80 °C freezer for the detection of inhaled allergen IgE and peripheral blood IL-36 family cytokine levels.

#### Detection of serum IL-36 family cytokine levels

An enzyme-linked immunosorbent assay (ELISA) was used to detect IL-36 family cytokine concentration levels in the serum of all subjects. The ELISA test kit was manufactured by Shanghai YOBIBO Trading Co., Ltd. The intraplate coefficient of variation was < 10%, the sensitivity of IL-36α detection was < 310 pg/ml, the sensitivity of IL-36β detection was < 3.1 pg/ml, the sensitivity of IL-36γ detection was < 9.3 pg/ml, the sensitivity of IL-36Ra detection was < 94 pg/ml, and the sensitivity of IL-38 detection was < 12.5 pg/ml. The OD value of each well was measured by placing a 96-well plate in a microplate reader at a wavelength of 450 nm, and the target protein concentration was directly proportional to the OD value. The concentration of target protein in the sample was calculated by plotting the standard curve, and duplicate wells were set for the determination of IL-36 family cytokine levels in each sample. The final data of IL-36 family cytokine levels in each sample were the mean value of duplicate wells. The experiment was performed by the same experimental operator at room temperature in strict accordance with the instructions.

#### 22-item sino-nasal outcome test (SNOT-22) score

Patients performed an assessment based on 22 bothersomeness problems associated with AR symptoms and quality of life that occurred in the preceding week and included 3 dimensions: physical problems, functional limitations, and emotional outcomes, each with 0 to 5 points, with “0” representing no issues and “5” representing very severe issues, for a total score of 0 to 110 points.

#### Severity of illness visual analogue scale (VAS) score

Patients underwent a VAS assessment of the overall extent of their symptoms at the onset of AR over the preceding week, which included 12 assessment items: nasal itching, sneezing, runny nose, nasal congestion, ocular itching, lacrimation, eye redness, eye pain, cough, suffocation, wheezing, and squeezing sensation. Scores of 0 to 10 on the VAS scale indicated how bothersome these symptoms were for the patient, with “0” being not bothersome and “10” being extremely bothersome. The more severe the symptoms were, the higher the score, and the total score ranged from 0 to 120.

#### Detection of serum inhalant allergen IgE

The assay was performed by using a Blotray 866 automatic blotting instrument in accordance with the kit instructions. The allergen types included house dust, dust mite combination (house dust mite/dust mite), tree pollen combination (cypress/elm/sycamore/willow/poplar), grass combination (bitter wormwood/Artemisia/ragweed), mold combination (Penicillium punctatum/Mycosphaerella/Aspergillusfumigatus/Crossstreptomycetes/Rhizopus/Trichoderma), and animal fur dander combination (cat fur dander/dog fur dander). The sIgE test results were classified into grades 0–5 according to the concentration: grade 0, < 0.35 IU/mL; grade 1, 0.35 ≤ sIgE < 0.70 IU/mL; grade 2, 0.70 ≤ sIgE < 3.50 IU/mL; grade 3, 3.50 ≤ sIgE < 17.50 IU/mL; grade 4, 17.50 ≤ sIgE < 50.00 IU/mL; grade 5, sIgE ≥ 50.00 IU/mL. A score of ≥ 0.35 IU/mL was judged as positive, and grade 0 was judged normal. Positive results of serum allergen total immunoglobulin E (tIgE) were judged based on the following: age < 3 years, tIgE ≥ 20 IU/mL; age 3–6 years, tIgE ≥ 35 IU/mL; age 6–20 years, tIgE ≥ 51 IU/mL; age > 20 years, tIgE ≥ 100 IU/mL.

### Statistical analysis

SPSS 26.0 software was used for statistical processing, and the Shapiro-Wilk test was used to test the normality of the distribution of the obtained measurement data. The measurement data that exhibited a normal distribution were described by the mean ± standard deviation, and the measurement data that did not exhibit a normal distribution were expressed by the median and interquartile range (IQR). The independent sample *t* test or Mann-Whitney *U* test was used for comparisons between two groups; the Kruskal-Wallis *H* test was used for comparisons between groups for multiple independent samples, and the Bonferroni method was used for pairwise comparisons with statistically significant differences; Spearman rank correlation and Point-biserial correlation were used for correlation tests. Results with *P* < 0.05 were considered statistically significant.

## Results

### Study population

The baseline characteristics of the 77 patients with AR included in our analysis are shown in Table 1. Overall, a higher proportion of patients included had no history of smoking and had been working indoors for a long time. Moreover, most of the patients in this study had a 1- to 5-year history of AR-related symptoms (nasal itching, sneezing, runny nose, and nasal congestion).

**Table 1:** Baseline characteristics of the patients with AR (n = 77)

	Patients with AR
Mean age, years, median (IQR)	24 (17, 33)
Gender, n (%)	
Male	33 (42.86)
Female	44 (57.14)
With history of smoking, n (%)	
Yes	24 (31.17)
No	53 (68.83)
Working environment, n (%)	
Indoors	58 (75.32)
Outdoors	10 (12.99)
Both^a^	9 (11.69)
With history of AR, n (%)	
Yes	26 (33.77)
No	23 (29.87)
Undefined^b^	28 (36.36)
Time of AR-related symptoms, n (%)	
<1 year	14 (18.18)
1–<5 years	31 (40.26)
5–<10 years	19 (24.68)
≥ 10 years	13 (16.88)

*AR* allergic rhinitis, *IQR* interquartile range. ^a^ Patients with AR appear to work indoors for the same amount of time as they do outdoors. ^b^ Patients with AR did not know whether they had AR in the past. The data about age that did not conform to a normal distribution was expressed by the median and interquartile range

### Concentration levels and correlation analysis of IL-36 family cytokines in the peripheral blood of patients with AR

#### Concentration levels of IL-36 family cytokines in the peripheral blood of patients with AR

**Fig. 1:**
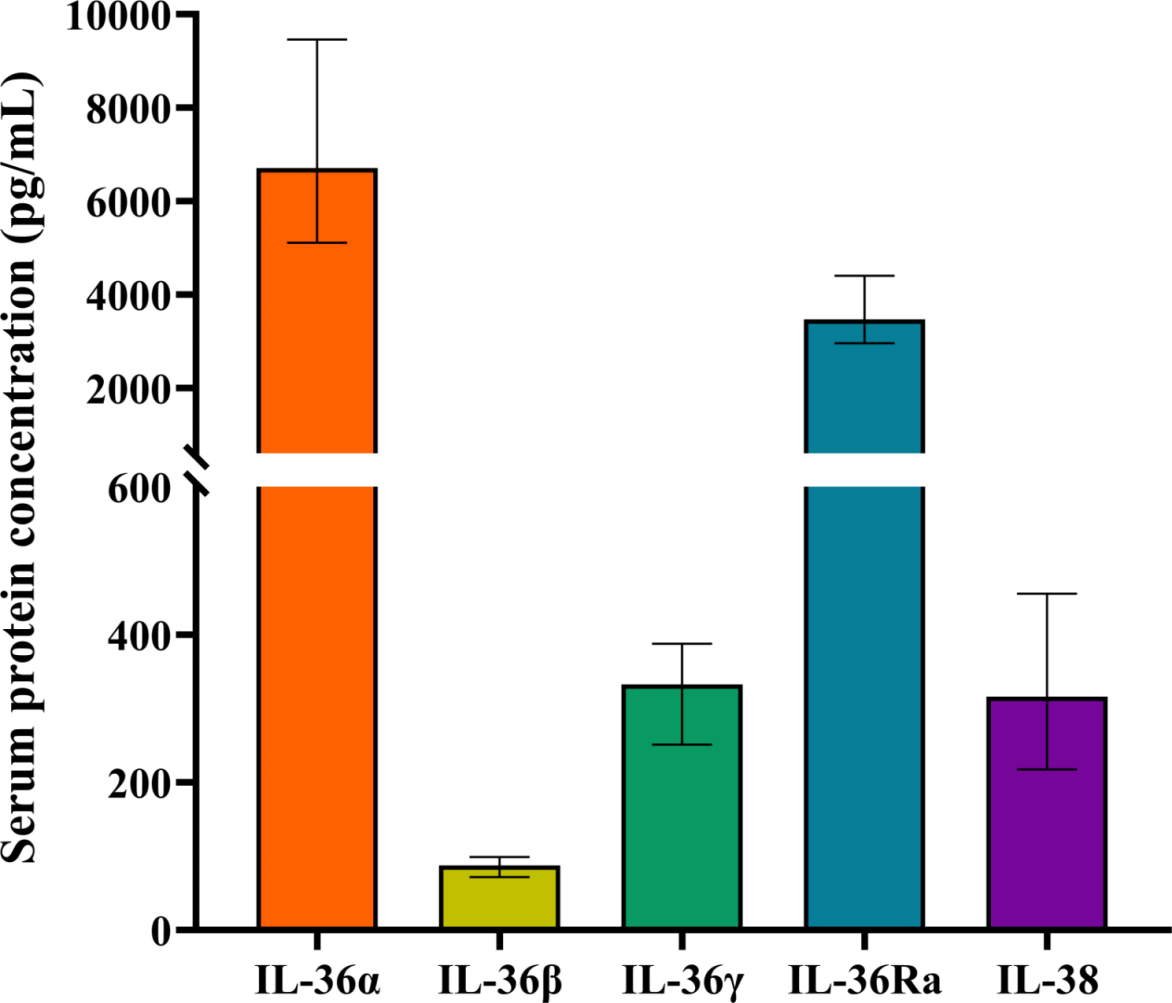
Concentrations of IL-36 family cytokines in the peripheral blood of 77 patients with AR. The data tested did not exhibit a normal distribution and were, therefore, described using the median and IQR. In patients with AR, the peripheral blood IL-36α concentration was highest, with a median of 6.71 × 10^3^ pg/mL (IQR: 5.12 × 10^3^, 9.46 × 10^3^); the IL-36β concentration was lowest, with a median of 87.86 pg/mL (IQR: 71.92, 99.41); the IL-36γ concentration was 332.79 pg/mL (IQR: 251.43, 387.65); the IL-36Ra concentration was 3.47 × 10^3^ pg/mL (IQR: 2.96 × 10^3^, 4.40 × 10^3^); and the IL-38 concentration was 316.18 pg/mL (IQR: 217.77, 455.62). *IL* interleukin; *AR* allergic rhinitis; *IQR* interquartile range

In the peripheral blood of patients with AR, the IL-36α concentration was highest, followed by IL-36Ra, IL-36γ, and IL-38, while IL-36β showed the lowest level (Fig. 1). Further comparisons between cytokines using the Kruskal-Wallis *H* test revealed that there were significant differences in concentration between cytokines (*P* < 0.01), except IL-36γ and IL-38.

#### Comparison of IL-36 family cytokine concentrations in the peripheral blood of patients with AR of different sexes and ages

Seventy-seven patients with AR were divided into a male group, a female group, a juvenile group (< 18 years) and an adult group (≥ 18 years) according to sex and age. The Mann-Whitney *U* test was used to analyze and compare the concentrations of IL-36 family cytokines in different groups. The results showed that the concentration level of IL-36α in the peripheral blood of juvenile patients was higher than that of adult patients, and the difference was statistically significant (*P* = 0.008), while there was no significant difference in the concentration of other cytokines of the IL-36 family between different sex and age groups (*P* > 0.05) (Table 2). We further used Point-biserial correlation and Spearman correlation to analyze the correlation between sex and age and IL-36 family cytokines, respectively. The results showed that neither sex nor age was significantly correlated with IL-36 family cytokines (*P* > 0.05). Therefore, sex and age were not considered confounding variables in this article.

**Table 2:** Concentrations of IL-36 family cytokines in the peripheral blood of patients with AR of different sexes and ages (pg/mL)

	IL-36α	IL-36β	IL-36γ	IL-36Ra	IL-38
Sex					
Male	7.43 × 10^3^(5.05 × 10^3^, 9.56 × 10^3^)	84.43 ± 23.81	343.47(270.19, 380.40)	3.45 × 10^3^(3.02 × 10^3^, 4.23 × 10^3^)	256.14(135.13, 423.70)
Female	6.51 × 10^3^(5.16 × 10^3^, 9.43 × 10^3^)	91.27(74.43, 100.46)	306.22(229.21, 390.31)	3.62 × 10^3^(2.88 × 10^3^, 4.51 × 10^3^)	387.41 ± 195.59
*z* value	-0.04	-1.08	-0.65	-0.09	-1.90
*P* value	0.967	0.280	0.517	0.926	0.057
Age					
< 18 years	8.63 × 10^3^(7.02 × 10^3^, 1.19 × 10^4^)	82.22(70.06, 95.84)	307.04 ± 90.64	3.22 × 10^3^±1.08 × 10^3^	359.60 ± 233.20
≥ 18 years	6.13 × 10^3^(4.97 × 10^3^, 8.92 × 10^3^)	88.15(74.30, 100.22)	333.25(251.43, 388.18)	3.62 × 10^3^(3.02 × 10^3^, 4.55 × 10^3^)	312.55(224.47, 461.23)
*z* value	-2.67	-0.68	-0.49	-1.70	-0.02
*P* value	**0.008**	0.497	0.626	0.090	0.981

*IL* interleukin, *AR* allergic rhinitis. The data was described by the mean ± standard deviation or the median and interquartile range. Mann-Whitney *U* test was used for comparison between two groups. *P* < 0.05 was considered significant. Bold text indicates that the difference is statistically significant

#### Correlation analysis between IL-36 family cytokines in peripheral blood

**Fig. 2:**
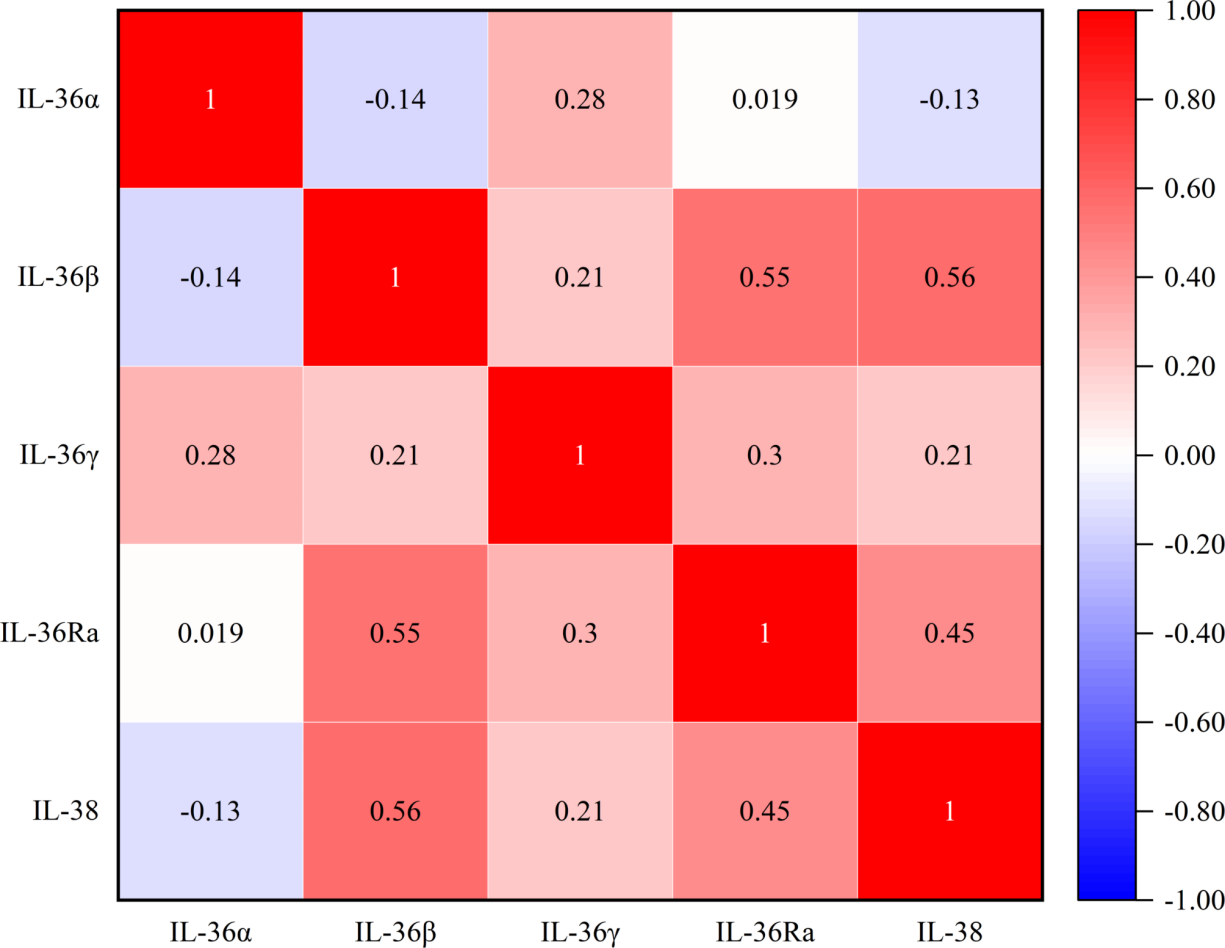
Correlation analysis of IL-36 family cytokine concentration levels in the peripheral blood of 77 patients with AR. The tested data did not exhibit a normal distribution, so Spearman rank correlation analysis was used. The results showed that the IL-36α level was positively correlated with IL-36γ level (*r* = 0.28, *P* = 0.013), the IL-36β level was positively correlated with IL-36Ra and IL-38 levels (*r* = 0.55, *P* < 0.001; *r* = 0.56, *P* < 0.001), the IL-36γ level was positively correlated with IL-36Ra level (*r* = 0.30, *P* = 0.008), the IL-36Ra level was positively correlated with IL-38 level (*r* = 0.45, *P* < 0.001), and there was no correlation between the levels of the remaining cytokines (*P* > 0.05). *IL* interleukin; *AR* allergic rhinitis

Spearman rank correlation analysis was used to analyze the correlation between IL-36 family cytokines in peripheral blood, and the results showed that the IL-36α level in peripheral blood was positively correlated with IL-36γ level with a correlation coefficient of 0.28 (*P* = 0.013); the IL-36β level was positively correlated with IL-36Ra and IL-38 levels with correlation coefficients of 0.55 (*P* < 0.001) and 0.56 (*P* < 0.001), respectively; the IL-36γ level was positively correlated with IL-36Ra level with a correlation coefficient of 0.30 (*P* = 0.008); the IL-36Ra level was positively correlated with IL-38 level with a correlation coefficient of 0.45 (*P* < 0.001); and there was no correlation between other cytokine levels (*P* > 0.05) (Fig. 2).

### SNOT-22 and VAS scores

Seventy-seven patients with AR underwent SNOT-22 scoring, and the median was 35 (IQR: 22, 49). VAS scores were calculated separately for nasal, ocular, and asthma-related symptoms in patients with AR. The total VAS score for nasal symptoms was defined as the sum of the VAS scores for the four symptoms of nasal itching, sneezing, runny nose and nasal congestion; the total VAS score for ocular symptoms was defined as the sum of the VAS scores for the four symptoms of ocular itching, lacrimation, eye redness and eye pain; the total VAS score for asthma-related symptoms was defined as the sum of the VAS scores for the four symptoms of cough, suffocation, wheezing, and squeezing sensation. The median total VAS score for nasal symptoms was 25 (IQR: 21, 29), the median VAS score for nasal itching symptom was 5 (IQR: 3, 8), the median VAS score for sneezing symptom was 8 (IQR: 5, 9), the median VAS score for runny nose symptom was 7 (IQR: 5, 9), and the median VAS score for nasal congestion symptom was 6 (IQR: 3, 8). The median total VAS score for ocular symptoms was 7 (IQR: 3, 12.50), the median VAS score for ocular itching symptom was 2 (IQR: 0.50, 5), the median VAS score for lacrimation symptom was 2 (IQR: 0, 4), the median VAS score for ocular redness symptom was 1 (IQR: 0, 3.50), and the median VAS score for eye pain symptom was 0 (IQR: 0, 2). The median total VAS score for asthma-related symptoms was 4 (IQR: 1, 10.50), the median VAS score for cough symptom was 1 (IQR: 0, 4), the median VAS score for suffocation symptom was 0 (IQR: 0, 3.50), the median VAS score for wheezing was 0 (IQR: 0, 2), and the median VAS score for squeezing was 0 (IQR: 0, 3).

### Overall distribution of inhaled allergens

Of 77 patients with AR, 49 tested positive for allergen tIgE, yielding a positive rate of 63.64% (49/77). In the analysis of the number of positive inhaled allergens, 1 had the highest number of positive inhaled allergens and 4 or more had the lowest number (Table 3); the highest positive intensity of allergen sIgE had the highest number of grade 1 and the lowest number of grade 3 and 4 (Table 3). Analysis of inhaled allergen-positive types revealed that 53 enrolled patients were allergic to perennial inhalant allergens (house dust, Dermatophagoides pteronyssinus/Dermatophagoides farinae, cat dander/dog dander), 14 enrolled patients were allergic to seasonal inhalant allergens (cypress/elm/sycamore/willow/poplar, wormwood/ragweed, Penicillium punctatum/Cladosporium fumigatus/Aspergillus fumigatus/Alternaria alternata/Rhizopus/Mucor), and only 10 enrolled patients were allergic to both perennial allergens and seasonal allergens.

**Table 3:** Distribution of the number of positive inhaled allergens and the highest positive intensity of allergen sIgE in 77 patients with AR

	Number	Percentage
Number of positive inhaled allergens		
1	46	59.74%
2	24	31.17%
3	4	5.19%
≥ 4	3	3.90%
The highest positive intensity of allergen sIgE		
Grade 1	41	53.25%
Grade 2	13	16.88%
Grade 3	5	6.49%
Grade 4	5	6.49%
Grade 5	13	16.88%

*sIgE* specific immunoglobulin E, *AR* allergic rhinitis

### Analysis of the correlation between IL-36 family cytokine levels and subjective and objective assessment results of patients with AR

#### Analysis of the correlation between IL-36 family cytokine levels and VAS scores and SNOT-22 score

Spearman rank correlation analysis showed that there was no significant correlation between the concentration levels of IL-36 family cytokines in peripheral serum and SNOT-22 score, the total VAS score for nasal symptoms or the total VAS score for asthma-related symptoms in patients with AR (*P* > 0.05), while there was a positive correlation between the concentration level of IL-36α in peripheral serum and VAS score for nasal congestion symptom with a correlation coefficient of 0.26, and the difference was statistically significant (*P* = 0.023), indicating that the higher the concentration levels of IL-36α in peripheral blood of patients with AR, the more prominent their nasal congestion symptom. There was a positive correlation between the concentration level of IL-36β in peripheral serum and the total VAS score for ocular symptoms with a correlation coefficient of 0.27, and the difference was statistically significant (*P* = 0.020). Specifically, there was a positive correlation between the level of IL-36β in peripheral blood and VAS scores for ocular itching and eye pain symptoms with correlation coefficients of 0.25 and 0.24, respectively. The differences were statistically significant (*P* = 0.028, *P* = 0.034), indicating that the higher the levels of IL-36β in the peripheral blood of patients with AR, the more severe the symptoms of ocular itching and eye pain, and even the overall symptoms of the eyes; there was no correlation between the other members of IL-36 family cytokines and the VAS scores of patients’ symptoms (*P* > 0.05) (Table 4).

**Table 4:** Correlation analysis of IL-36 family cytokine concentration levels with VAS scores and SNOT-22 score in peripheral blood of 77 patients with AR

Scoring items	IL-36α		IL-36β		IL-36γ		IL-36Ra		IL-38	
	*r* value	*p* value	*r* value	*p* value	*r* value	*p* value	*r* value	*p* value	*r* value	*p* value
Total VAS score for nasal symptoms	0.12	0.317	0.13	0.266	0.09	0.417	0.17	0.147	0.10	0.383
VAS score for nasal itching	0.00	0.996	0.11	0.360	-0.02	0.857	0.11	0.341	0.21	0.066
VAS score for sneezing	-0.10	0.401	-0.04	0.757	-0.05	0.693	0.00	0.983	-0.09	0.418
VAS score for runny nose	0.11	0.363	0.14	0.235	0.20	0.084	0.15	0.192	0.19	0.100
VAS score for nasal congestion	**0.26**	**0.023**	0.01	0.952	0.10	0.384	0.08	0.513	-0.11	0.333
Total VAS score for ocular symptoms	0.07	0.541	**0.27**	**0.020**	0.05	0.642	0.18	0.111	0.20	0.074
VAS score for ocular itching	0.04	0.746	**0.25**	**0.028**	-0.06	0.626	0.15	0.207	0.21	0.069
VAS score for lacrimation	0.04	0.715	0.12	0.299	0.08	0.502	0.06	0.624	0.10	0.370
VAS score for eye redness	0.04	0.732	0.13	0.276	0.06	0.611	0.20	0.084	0.00	0.999
VAS score for eye pain	0.12	0.280	**0.24**	**0.034**	0.02	0.849	0.14	0.216	0.18	0.120
Total VAS score for asthma-related symptoms	0.11	0.335	0.09	0.459	0.01	0.954	-0.09	0.445	0.00	0.994
VAS score for cough	0.21	0.068	0.12	0.299	0.03	0.794	-0.02	0.874	0.03	0.799
VAS score for suffocation	0.09	0.450	-0.05	0.677	0.20	0.088	-0.14	0.241	-0.03	0.807
VAS score for wheezing	-0.01	0.922	0.02	0.893	0.01	0.898	-0.06	0.575	-0.07	0.573
VAS score for squeezing	-0.10	0.409	0.14	0.241	-0.08	0.482	-0.03	0.798	0.07	0.530
SNOT-22 score	0.11	0.327	0.02	0.890	0.08	0.488	-0.06	0.624	-0.12	0.308

*IL* interleukin, *VAS* visual analogue scale, *SNOT-22* 22-item sino-nasal outcome test, *AR* allergic rhinitis. The spearman correlation analysis method was used for analysis. *P* < 0.05 was considered significant. Bold text indicates that the difference is statistically significant and there is a correlation between the two indicators

#### Comparison of IL-36 family cytokine concentration levels in peripheral blood between patients with positive and negative inhaled allergen tIgE

The two independent samples *t* test or Mann-Whitney *U* test was used to compare the concentration differences of IL-36 family cytokines between the inhaled allergen tIgE-positive and tIgE-negative groups, and it was found that there were no significant differences in the concentration levels of IL-36α, IL-36β, IL-36γ, IL-36Ra, and IL-38 between the two groups (*P* > 0.05), indicating that tIgE positivity did not affect the concentrations of IL-36 family cytokines in the peripheral blood of patients with AR (Table 5).

**Table 5:** Comparison of IL-36 family cytokine concentration levels in peripheral blood between patients with positive and negative inhaled allergen tIgE (pg/mL)

IL-36 family cytokines	tIgE positive group	tIgE negative group	*z/t* value	*P* value
IL-36α	6.84 × 10^3^(5.38 × 10^3^, 1.03 × 10^4^)	6.01 × 10^3^(4.89 × 10^3^, 8.86 × 10^3^)	-1.31	0.189
IL-36β	88.52 ± 23.66	80.93 ± 34.66	-1.23	0.219
IL-36γ	343.47(262.96, 392.82)	306.00(249.98, 379.55)	-0.88	0.379
IL-36Ra	3.50 × 10^3^(3.15 × 10^3^, 4.43 × 10^3^)	3.40 × 10^3^(2.62 × 10^3^, 4.38 × 10^3^)	-0.89	0.374
IL-38	343.87(229.00, 489.95)	270.51(200.40, 415.26)	-1.03	0.304

*IL* interleukin, *tIgE* total immunoglobulin E. The data was described by the mean ± standard deviation or the median and interquartile range. The independent sample *t* test was used for normal distribution data, otherwise Mann-Whitney *U* test was used for comparison between tIgE positive group and tIgE negative group. *P* < 0.05 was considered significant

#### Correlation analysis of IL-36 family cytokine concentrations in peripheral blood with the number of positive inhaled allergens and the highest positive intensity of allergen sIgE

Spearman rank correlation analysis revealed that there was no correlation between IL-36 family cytokine levels in peripheral blood and the number of positive inhaled allergens or the highest positive intensity of allergen sIgE in patients with AR (*P* > 0.05) (Table 6).

**Table 6:** Correlation analysis of IL-36 family cytokine concentrations in peripheral blood with the number of positive inhaled allergens and the highest positive intensity of allergen sIgE

IL-36 family cytokines	Number of positive inhaled allergens			The highest positive intensity of allergen sIgE	
	*r* value	*P* value		*r* value	*P* value
IL-36α	0.18	0.127		0.13	0.268
IL-36β	-0.10	0.401		-0.09	0.411
IL-36γ	-0.07	0.546		-0.08	0.504
IL-36Ra	-0.01	0.951		0.03	0.767
IL-38	-0.12	0.292		-0.09	0.426

*IL* interleukin, *sIgE* specific immunoglobulin E. The spearman correlation analysis method was used for analysis. *P* < 0.05 was considered significant

#### Comparison of differences in IL-36 family cytokine concentrations in peripheral blood between the perennial allergen group, seasonal allergen group and mixed allergen group

**Fig. 3:**
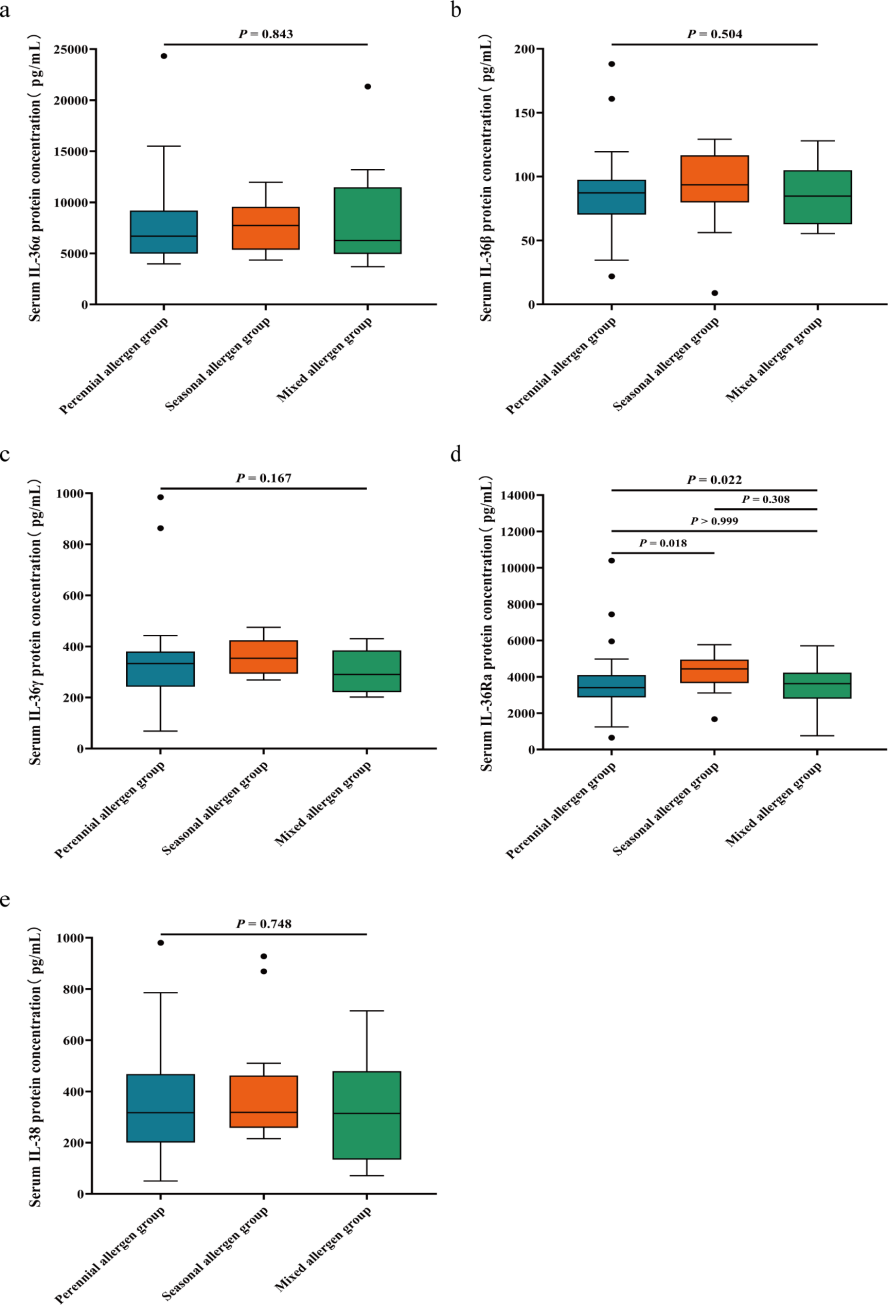
Patients with AR were divided into three groups according to different types of allergen positivity: perennial allergen group, seasonal allergen group and mixed allergen group. The test data did not conform to a normal distribution, so the Kruskal-Wallis *H* test was used to compare the differences in the levels of IL-36 family cytokines among the three groups, and the Bonferroni method was further used for pairwise comparison if the differences were statistically significant. (**a**) There was no significant difference in peripheral blood IL-36α concentration among the three groups of patients with AR; (**b**) There was no significant difference in peripheral blood IL-36β concentration among the three groups of patients with AR; (**c**) There was no significant difference in peripheral blood IL-36γ concentration among the three groups of patients with AR; (**d**) Peripheral blood IL-36Ra concentration in patients with AR in the seasonal allergen group was higher than that in the perennial allergen group with a statistically significant difference (*P* = 0.018), and there was no significant difference in peripheral blood IL-36Ra concentration between patients with AR in the mixed allergen group and those in the perennial allergen group and seasonal allergen group; (**e**) There was no significant difference in peripheral blood IL-38 concentration among the three groups of patients with AR. *AR* allergic rhinitis; *IL* interleukin

According to inhalant allergen-positive types in patients with AR, 77 patients with AR were divided into 3 groups: allergic to perennial inhalant allergens only—perennial allergen group (53 cases), allergic to seasonal inhalant allergens only—seasonal allergen group (14 cases), and allergic to both perennial allergens and seasonal allergens—mixed allergen group (10 cases). By comparing the concentration levels of IL-36 family cytokines among the three groups, IL-36α, IL-36β, IL-36γ and IL-38 levels were found to be not significantly different among the three groups (*P* > 0.05). The concentration levels of IL-36Ra were significantly different among the three groups (*P* = 0.022), and further pairwise comparisons revealed that there was a difference in IL-36Ra levels between the perennial allergen group and the seasonal allergen group (*P* = 0.018), indicating that IL-36Ra levels in peripheral blood were higher in patients with AR with seasonal allergen allergy than in perennial allergen allergic patients (Table 7) (Fig. 3).

**Table 7:** Comparison of IL-36 family cytokine concentration differences in peripheral blood among the perennial allergen group, seasonal allergen group and mixed allergen group (pg/mL)

	IL-36α	IL-36β	IL-36γ	IL-36Ra	IL-38
Perennial allergen group	6.68 × 10^3^(4.98 × 10^3^, 9.19 × 10^3^)	87.26(70.28, 97.58)	332.79(242.25, 380.40)	3.41 × 10^3^(2.87 × 10^3^, 4.10 × 10^3^)	317.06(200.48, 468.07)
Seasonal allergen group	7.66 × 10^3^±2.24 × 10^3^	90.10 ± 30.65	362.99 ± 68.81	4.24 × 10^3^±1.05 × 10^3^	317.95(257.88, 462.72)
Mixed allergen group	6.26 × 10^3^(4.94 × 10^3^, 1.15 × 10^4^)	86.09 ± 23.57	303.73 ± 86.61	3.53 × 10^3^±1.32 × 10^3^	326.73 ± 209.09
* h* value	0.34	1.37	3.57	7.60	0.58
*P* value	0.843	0.504	0.167	**0.022**	0.748

*IL* interleukin. The data was described by the mean ± standard deviation or the median and interquartile range. Kruskal-Wallis *H* test was used for comparison among for three independent groups. *P* < 0.05 was considered significant. Bold text indicates that the difference is statistically significant

## Discussion

Recent studies suggest that IL-36 family cytokines are highly expressed in a variety of inflammatory diseases, such as CRS, asthma and AD, and are closely related to the occurrence and development of disease [11–15, 18]. It was reported that [16, 19] in patients with AR, the protein concentrations of IL-36α, IL-36β, IL-36γ, IL-36Ra and IL-38 in peripheral blood were significantly higher than those in healthy controls, with IL-36γ having the highest concentration. In contrast, the highest level of IL-36α and the lowest level of IL-36β were found in the peripheral blood of patients with AR in this study, which may be related to the fact that the samples included in the two studies were in different regions or disease courses. This study also found that there was no obvious sex difference in IL-36 family cytokine levels, but the concentration levels of IL-36α in the peripheral blood of patients in the adolescent group were higher than that of patients in the adult group, which may be related to differences in common allergens and immune response status between the two groups. Variations may range from different allergens stimulating the nasal mucosa to other factors affecting synthesis, secretion and activation of the various cytokines and inflammatory proteins in the body; there are a series of complex pathways involved that are affected by genetics, environment and other factors, and the specific mechanisms are currently unclear. Future studies with large samples are needed.

The results of this study showed a weak positive correlation between IL-36α levels and IL-36γ levels in the peripheral blood of patients with AR (*r* = 0.28, *P* = 0.013). In addition, this study revealed that the IL-36β level in peripheral blood was moderately correlated with the level of the IL-36R antagonist IL-36Ra (*r* = 0.55, *P* < 0.001), and IL-38 (*r* = 0.56, *P* < 0.001). There was also a weak positive correlation between peripheral blood IL-36γ level and IL-36Ra level (*r* = 0.30, *P* = 0.008). It is believed that [20] IL-38 and IL-36Ra are not typical receptor antagonists, and their inhibitory effect weakens with increasing concentrations; they may even exert proinflammatory effects as agonists at high concentrations (IL-38 level > 250 ng/mL) [21]. The median IL-38 concentration level in this study was 316.18 pg/mL (IQR: 217.77, 455.62), which is not in a high concentration. Therefore, this study caused us to speculate that while IL-36α, IL-36β and IL-36γ promote the inflammatory response in AR, the body forms a positive feedback pathway to antagonize this severe inflammatory response and maintain balance, thereby promoting the production of IL-36Ra and IL-38. However, during this process, IL-38 and IL-36Ra may always play a weak inhibitory role, which is an idea that is similar to the suggestion from Chu et al. [22] that the increase in inflammatory cytokine levels can trigger the release of IL-38 even though its immunoregulatory effect may not be sufficient to counteract the cytokine storm. Interestingly, the IL-36Ra level in peripheral blood was found to be positively correlated with IL-38 level in this study (*r* = 0.45, *P* < 0.001), while several studies have shown that IL-36Ra functions as a receptor antagonist and does not share the same pathway [23–25]. IL-36Ra binds IL-36R to mediate its antagonism by inhibiting the recruitment of IL-1RAcP and the dimerization of IL-36R/IL-1RAcP [24, 25]. IL-38, by binding to IL-36R, may have antagonistic effects by recruiting one of the inhibitory coreceptors of the IL-1R family, SIGIRR, TIGIRR1, and/or TIGIRR2 [23]. Therefore, based on this study, we speculate that IL-36Ra and IL-38 may assist each other when they act as inhibitors of the inflammatory response, but the specific mechanism needs to be further studied.

In this study, we found a positive correlation between peripheral blood IL-36α level and VAS score for nasal congestion symptom in patients with AR (*r* = 0.26, *P* = 0.023). It has been shown that [15] IL-36α, IL-36β and IL-36γ stimulate nasal mucosal vascular endothelial cells to cause increased permeability in patients with CRS, while in AR, an inflammatory disease of the nasal mucosa with a similar inflammatory pattern, IL-36α may also cause nasal congestion by acting on nasal mucosal vascular endothelial cells to increase vascular permeability, exudate plasma and inflammatory substances, and form local mucosal edema. It has also been documented that [19] IL-36α promotes Th17-type inflammation via the PI3K/AKT and ERK pathways, while IL-17 and IL-23 promote IL-36α production, thus forming a positive feedback loop. Anti-IL-36α treatment significantly attenuates Th17 responses in AR mice, and the results of this study also confirm that IL-36α plays an important role in the AR disease process. In addition, this study found a weak positive correlation between peripheral blood IL-36β level and the total VAS score for ocular symptoms and VAS scores for ocular itching and eye pain symptoms in patients with AR (*r =* 0.27, *P* = 0.020; *r =* 0.25, *P* = 0.028; *r =* 0.24, *P* = 0.034), suggesting that IL-36β may be involved as a proinflammatory cytokine in the pathogenesis of ocular itching and eye pain symptoms in patients with AR, but the specific mechanism of action is still unclear. Recent studies suggest that asthma and AR share common and interrelated epidemiological, clinical, and inflammatory mechanisms [26], and IL-36 family cytokines also play an important role in the development of asthma [12, 27]. Four asthma-related symptoms, namely, cough, suffocation, wheezing, and squeezing, were included in the assessment of the severity of illness through the VAS score items in this study, and unfortunately, no correlation was observed between IL-36 family cytokine levels and the VAS scores of asthma-related symptoms. This may be because AR often precedes asthma-related symptoms and is a risk factor for asthma; approximately 15–38% of patients with AR experience asthma-related symptoms [28], while only a few of the study subjects included in this study developed related symptoms. Interestingly, the results of this study also showed differences in peripheral blood IL-36Ra concentration between patients with perennial allergen allergies and seasonal allergen allergies; there was a higher concentration in the seasonal allergen allergy group, indicating that allergen species can influence peripheral blood IL-36Ra concentration in patients with AR.

In summary, IL-36α and IL-36β levels were correlated with the severity of AR and could be used as objective indicators to assess the severity of AR. However, this study was only exploratory and aimed to discover some possible or potential correlation results, so we did not perform correction for multiple testing, and our team will conduct further research in this area in the future. In addition, in this study, only IL-36 family cytokine levels were correlated with subjective and objective AR assessment results, and the specific mechanism of action of IL-36 family cytokines in AR was not investigated. Their mechanism of action in AR was only inferred based on ideas from the literature, and their specific mechanism of action in AR needs to be further investigated in the future.

## Conclusion

IL-36α level was positively correlated with the VAS score for nasal congestion symptom, and IL-36β level was positively correlated with the total VAS score for ocular symptoms and the VAS scores for ocular itching and eye pain symptoms. Overall, these findings indicated that the protein concentration levels of IL-36α and IL-36β cytokines in peripheral blood were correlated with the severity of the condition of patients with AR, and IL-36α and IL-36β may be used as objective indicators to evaluate the severity of the condition of patients with AR.

## Data Availability

The datasets used and/or analyzed during the current study are available from the corresponding author on reasonable request.

## Abbreviations

AR: Allergic rhinitis
IL: Interleukin
ELISA: Enzyme-linked immunosorbent assay
SNOT-22: 22-item sino-nasal outcome test
VAS: Visual analogue scale
IgE: Immunoglobulin E
sIgE: Specific immunoglobulin E
tIgE: Total immunoglobulin E
IL-36R: IL-36 receptor
IL-1RAcP: Interleukin-1 receptor accessory protein
MyD88: Myeloid differentiation response gene 88
NF-κB: Nuclear factor-κB
MAKP: Mitogen-activated protein kinase
AD: Atopic dermatitis
CRSwNP: Chronic rhinosinusitis with nasal polyps
CRS: Chronic rhinosinusitis
TLR: Toll-like receptor
ARIA: Allergic Rhinitis and its Impact on Asthma
IQR: Interquartile range
